# Application and Evaluation of the Flipped Classroom Based on Micro-Video Class in Pharmacology Teaching

**DOI:** 10.3389/fpubh.2022.838900

**Published:** 2022-03-24

**Authors:** Yi-Ying Wu, Sha Liu, Qiong Man, Feng-Lin Luo, Ya-Xin Zheng, Sheng Yang, Xin Ming, Fang-Yan Zhang

**Affiliations:** Department of Pharmacology, School of Pharmacy, Chengdu Medical College, Chengdu, China

**Keywords:** flipped classroom, pharmacology, medical education, feedback questionnaire, micro-video class

## Abstract

With the continuous development of information technology, new teaching resources “micro-video class” and teaching model “flipped classroom” have gradually attracted the attention of teachers. Whether and how they can be applied in pharmacology teaching has already become the focus of medical education research in recent years. This paper explores the application and evaluation of the flipped classroom based on micro-video class in pharmacology teaching in our college. Students in Class 1 and Class 2 majoring in clinical medicine of 2018 in Chengdu Medical College were randomly divided into experimental group and control group. The teaching model of flipped classroom based on micro-video class was used in the experimental group, while the traditional teaching model was used in the control group. Theory tests and questionnaires were carried out at the end of the course. The average scores of theoretical knowledge in experimental group were significantly higher than those in control group (*P* < 0.05). In addition, the results of the feedback questionnaire showed that the overall satisfaction of students participating in flipped classroom based on micro-video class was higher (*P* < 0.05), and students thought that their learning enthusiasm, learning efficiency, and abilities of autonomous learning and problem-solving were greatly improved compared with those of students taught applying the traditional teaching model. Flipped classroom based on micro-video class model successfully improved the outcome of pharmacology teaching. It is supposed to provide reference for the reform of pharmacology teaching in medical college.

## Introduction

With the rapid development of science and technology, society puts higher and higher demand for college students' abilities in all aspects. Educators pay more attention to the teaching effect and quality of colleges and universities. At present, the main problem of college teaching is that the traditional passive teaching mode is not conducive to cultivate the innovative thinking and autonomous learning ability of students. Therefore, flipped classroom is more and more applied to traditional teaching and widely recognized by students. This teaching method is constructively based on the student-centered teaching concept, and gives full play to students' subjective initiative. The basic premise of flipped classroom is require students to self-study lesson content before class, so that classroom time can be devoted to teaching and learning activities (1, 2). However, it is difficult for students to achieve the teaching requirements only by self-study with textbooks. To overcome these issues, educational researchers have come up with an innovative teaching model that combines micro-video class with flipped classroom (3).

Micro-video class is to explain a certain knowledge point (key points, difficulties or doubts) with online short video. As the basic courseware resources of screen class, micro-video class has been welcomed by most college students because of its short and concise characteristics. And the new teaching model of flipped classroom based on micro-video class has been widely used in many medicine and pharmacy subjects (4–6). Pharmacology is a main compulsory course in clinical medicine and pharmacy majors. However, many students think that Pharmacology is boring and difficult to study (7). In order to improve the teaching effect of pharmacology, a flippedclassroom based on micro-video class teaching model for pharmacology course has been designed and implemented in clinical medicine major in our school. And the teaching effect of micro-video class combined flipped classroom was evaluated by the theoretical examination and questionnaire survey, and compared with traditional teaching model.

## Study Participants and Method

### Study Participants

The study is conducted by the School of pharmacy at Chengdu Medical College. The subjects are two parallel classes of senior undergraduates majoring in Clinical Medicine (class of 2018). 73 students from Class 1 are divided into experimental group (flipped classroom based on micro-video class), and 85 students from Class 2 are divided into control group (Traditional classroom). The students from the two classes are evenly assigned to parallel classes based on their college entrance examination scores, and take the same courses throughout college. All of the procedures in this study are approved by the Ethics Committee of Chengdu Medical College, and informed consent is obtained from the students.

### Teaching Methodology

Both the control group and the experimental group are taught by the same teacher, and the teaching contents and periods of the two groups are the same.

#### Design of the Flipped Classroom Based on Micro-Video Class Teaching Model

The experimental group adopt the teaching method of micro-video class combined with flipped classroom.ive is formulated for each session. 3 class h (120 min) are allocated to each flipped classroom to complete a teaching content. A total of 13 knowledge points are made into micro class videos (Table 1).

**Table 1:** Teaching contents and micro class videos.

**Chapter**	**Teaching content**	**Micro class video**	**Length of the video**
1	Diuretics	High efficacy diuretics	10 min
		Moderate efficacy diuretics	9 min
		Low efficacy diuretics	5 min
2	Antihypertensive drug	Hypertension overview and drug classification	11 min
		First-line antihypertensive drugs	10 min
3	Antianginal drug	Pathophysiology of angina pectoris	4 min
		Nitroglycerin	8 min
		Other antianginal drug	6 min
4	Antiarrhythmic drug	Overview of antiarrhythmic drugs	12 min
		Commonly used antiarrhythmic drugs	11 min
5	Drugs for heart failure	Pathophysiology of heart failure	9 minutes
		Cardiac glycosides	10 min
		Other drugs for heart failure	10 min

Before class:

The pharmacology courses reform was built on the Chaoxing Campus Online Teaching Platform. The teacher uploads micro class videos and teaching objectives to the online platform three days in advance, and requires students to learn before class. The online platform allows students to watch micro-videos and communicate with teachers at any time. At the same time, students' online self-learning is continuously supervised by the platform. And teachers can check it at any time and guide students to learn.

In class:

First of all, knowledge review: The teacher systematically summarizes the key and difficult knowledge points of students' autonomous learning before class.

Secondly, classroom interaction: This session is mainly carried out in the form of flipped classroom. Based on the learning contents of the pre-class video, the teacher sets up some questions and case analyses. Students work in groups to discuss and analyze the questions and cases raised by teacher, and use the theoretical knowledge they have mastered to answer and solve the problems. At the same time, the teacher fully mobilizes students to think about questions and cases, and guide students to find problems and solve them flexibly and correctly.

Finally, In-class quiz:In order to evaluate students' mastering degree of the learning content, the teacher sets a in-class test and requires students to complete it independently.

#### Design of the Traditional Classroom Teaching Model

The control group adopt the traditional teacher-centered model of teachers' lecturing basically and students' learning passively. Students are also required to preview the teaching contents before class by textbook. And the same cases are analyzed in the control class, but not in the form of flipped classroom.

### Effect Evaluation

#### The Theory Test

At the end of each teaching content, about 10 choice questions are selected for in-class quiz. Answering correctly sixty percent of the test questions will be considered as passed. Finally, we compared the average test scores between two groups of students under different teaching methods.

#### The Questionnaire Survey

At the end of the course, a on-site questionnaire is distributed to students in the form of online electronic questionnaire. The students fill in the questionnaire independently in an anonymous way. The questionnaire is designed based on validated questionnaires of previous studies (8, 9). And the responses are scored using a 5-point Likert scale (rang from one strongly disagree to five strongly agree) to evaluate teaching and learning methods. The reliability of the scale is checked by Cronbach's alpha coefficient.

### Statistical Analysis

The Wilcoxon signed-rank test is used to compare the questionnaire survey responses between the two groups. The distribution of the questionnaire score is skewed. The test score is presented as means ± standard deviation (SD) and analyzed by independent sample *T*-test. *P* < 0.05 is considered statistically significant. All statistics and data analyses are performed using SPSS 21.0 software (SPSS Inc., USA).

## Results

### Theory Test

As shown in Table 2, the average test score of experimental group (88.62 ± 2.65) was significantly higher than those of the control group (81.29 ± 2.79) (*P* < 0.05). Meanwhile, the test pass rate in the experimental group also increased, but no statistical difference was observed between the two groups (*P* > 0.05).

**Table 2:** The average score of theory examination.

**Groups**	**The number of students**	**Test score**	**Pass rate (%)**
Experimental group	73	88.62 ± 2.65^*^	89.59 ± 2.08
Control group	85	81.29 ± 2.79	86.35 ± 2.44

* *p < 0.05 vs. Control group*.

### Students' Questionnaire Results

158 copies of questionnaires were sent out, and 151 copies were received with a recovery rate of 95.57%. The questionnaire results were shown in Table 3. 84.06% of students in the experimental group were satisfied with the teaching and learning activities. And 79.71% of students in the experimental group agreed that flipped classroom based on micro-video class teaching method stimulated their learning interest in pharmacology. In addition, 89.86% of students in the experimental group thought that they could understand the teaching contents better, and more than 70% of students agreed that their autonomous learning, analyzing and problem-solving abilities were developed via flipped classroom based on micro-video class teaching model.

**Table 3:** Responses to questionnaire from students regarding the teaching and learning effects.

**Statement number**	**Statement**	**Experimental group (*n* = 69)**			**Control group (*n* = 82)**		
		**SA/A (%)**	**U (%)**	**D/SD (%)**	**SA/A (%)**	**U (%)**	**D/SD (%)**
Q1	You satisfied with the current teaching and learning model.	84.06	10.14	5.80	67.07	19.51	13.41
Q2	The current teaching and learning methods stimulate your learning interest in pharmacology.	79.71	13.04	7.25	47.56	30.49	21.85
Q3	The current teaching and learning methods improve the learning efficiency and enable you to understand rather than simply memorize the teaching content.	89.86	7.25	2.90	70.73	24.39	4.88
Q4	The current teaching and learning methods enhance your autonomous learning ability.	85.51	8.70	5.80	56.10	26.83	17.07
Q5	The current teaching and learning methods develop your ability to analyze and solve problems.	76.81	14.49	8.70	51.22	20.73	28.05

*Values are percentage of students.*

*SA, Strongly agree; A, Agree; U, uncertainty; D, Disagree; SD, Strongly disagree*.

Furthermore, the Wilcoxon test showed that higher scores on all the five questions were obtained in the experimental group than that of the control group (*P* < 0.01) (Table 4). Compared with traditional teaching class, the satisfaction degree of students participating in flipped classroom based on micro-video class is significantly improved (*P* < 0.01). Its benefits include stimulating learning interest (*P* < 0.001), improving learning efficiency (*P* < 0.01), enhancing abilities of autonomous learning (*P* < 0.001), analyzing and solving problems (*P* < 0.01).

**Table 4:** Comparison of course evaluation scores between flipped classroom based on micro-video class and traditional model.

**Statement number**		**Experimental group**	**Control group**	**z-value**	p^*^-value
Q1	n (missing)	73 (4)	85(3)	−2.639	0.008
	Mean ± SD	4.12 *± 0*.81	3.73 *± 0*.93		
	M (IQR)	4.00 (4.00, 5.00)	4.00 (3.00, 4.00)		
Q2	n (missing)	73 (4)	85 (3)	−4.052	0.000
	Mean ± SD	4.07 ±*0*.88	3.41 ± 1.01		
	M (IQR)	4.00 (4.00, 5.00)	3.00 (3.00, 4.00)		
Q3	n (missing)	73 (4)	85 (3)	−3.198	0.001
	Mean ± SD	4.41 ±*0*.75	3.98 ±*0*.87		
	M (IQR)	5.00 (4.00, 5.00)	4.00 (3.00, 5.00)		
Q4	n (missing)	73 (4)	85 (3)	−4.190	0.000
	Mean ± SD	4.26 ±*0*.85	3.57 ± 1.05		
	M (IQR)	4.00 (4.00, 5.00)	4.00 (3.00, 4.00)		
Q5	n (missing)	73 (4)	85 (3)	−3.296	0.001
	Mean ± SD	3.99 ± 0.99	3.33 ± 1.26		
	M (IQR)	4.00 (4.00, 5.00)	4.00 (2.00, 4.00)		

* *Based on Wilcoxon signed-rank test, P < 0.05 was considered significant*.

The above results revealed that the flipped classroom based on micro-video class teaching model was superior to the traditional teaching model in improving students' initiative and cultivating students' ability, and brought better teaching effect.

## Discussion

Pharmacology is a professional basic course for medical students, and also a discipline bridging basic medicine and clinical medicine. The course with boring and strong theoretical contents covers a wide range of disciplines (10). The traditional passive learning mode further reduces students' enthusiasm and initiative. As a result, students only want to cope with the exam, but can't actually understand and master the learning content, which is a major challenge to the teachers.

How to broaden teaching ideas and improve teaching methods to enhance the teaching effect is difficulty for every pharmacological educator. This study aims to explore how to apply micro-video class combined with flipped classroom model in pharmacology teaching to make the abstract and boring content vivid. So that students can activate thinking, give play to their subjective initiative, and improve learning effect. And the application effect of this teaching model is evaluated through theory test and questionnaire survey, and compared with the traditional teaching model. We select the chapters of drugs acting on the cardiovascular system as a pilot reform. These chapters are usually considered difficult for students to master and these drugs are commonly used in clinic. The results of theory tests indicated that the learning effect of students with flipped classroom based on micro-video class was significantly better than that of students with traditional teaching (*P* < 0.05). By self-learning the micro-video class through the online platform and efficiently participating in flipping classroom, students deeply understood and mastered the theory knowledge. Furthermore, the results of the teaching feedback questionnaire exerted that most of the students in experimental group satisfied with the micro-video class combined with flipped classroom teaching model, provided more positive evaluations and gave higher scores to the course (*P* < 0.05). Compared with traditional teaching classroom, the students who participated in flipped classroom based on micro-video class showed higher enthusiasm for learning in pharmacology, understood teaching contents more easily and remembered better. They believed that the novel teaching and learning model greatly stimulated their learning enthusiasm, developed their abilities in autonomous learning, analyzing and solving problems. It suggests that flipped classroom based on micro-video class teaching model is more recognized by students than traditional teaching model.

In previous studies, the teaching model of micro-video class combined with flipped classroom has been proved to achieve good teaching effects in several college courses, such as college English (11), Emergency Medicine (4), neurosciences (5), animal health (6). These studies indicate that flipped classroom effectively mobilizes students' learning ability and greatly improves the interaction between teaching and learning. Meanwhile, combined with the short online teaching videos before class, the shortage of class hours is effectively relieved, allowing class time to be more productively used for higher-level activities. Rather than providing answers directly, the teachers become a guider and a resource provider to help students arrive conclusions. This teaching model is more student-centered and gets more positive feedback from the students.

Our research shows that this teaching model is also effective in pharmacology teaching for clinical medical students. In pharmacology course, besides mastering basic concepts, acquiring pharmacotherapeutic skills and competencies also comprises the primary goal for clinical medical students. Flipped classroom based on micro-video class can help students achieve this goal. Since students have learned the basic theoretical knowledge of drugs through micro videos before class, the flipped class gives teachers more time for analyzing clinical cases, explaining how to use drugs safely and effectively, to help students establish clinical thinking. Students also make full use of classroom time for applied activities and learning, thus better gain competencies. At the end of the course reform, most students said that their understanding of drug interaction and clinical rational drug use had improved, and they were much more confident in discussing drug therapy with doctors in clinical clerkship teaching. In addition, the teacher also reported that the students in the experimental group were more active in class and asked more questions than those in the traditional teaching group. This novel teaching model enables students to demonstrate more comprehensive and critical thinking.

In general, the application of flipped classroom based on micro-video class in pharmacology course is beneficial to cultivate students' ability to comprehensively analyze and solve problems, enhance their autonomy and interest in learning.

Nonetheless, this teaching model is not without drawbacks. The main challenge is the increased pressure and preparation time for both teachers and students. For teachers, it is a large amount of time investment for designing and recording micro class videos, collecting clinical cases and designing creative classroom activities related to pharmacotherapeutic competencies to keep students' participation and interest. It is also very time-consuming to manage online teaching systems and supervise students' self-learning processes. In addition, students think more actively or have difficulties in self-learning, thus teachers need to spend more time answering questions outside class. Meanwhile, in order to meet the needs of current social development, teachers should not only be fully familiar with the theory, but also constantly update the teaching content and improve their teaching skills.

For students, it takes more time to self-study micro-video class, while flipped classes may increase their anxiety. In class, teachers review the knowledge points of the online micro videos through question-and-answer, rather than direct explanation. Moreover, a large amount of classroom time is used for student-centered activities. In order to participate in activities more effectively and pass the course assessment, students have to spend more time preparing outside class and thus feel anxious. This is why some students were dissatisfied with this new teaching mode at the beginning and were more accustomed to passive learning in traditional teaching. However, with the increasing participation in classroom activities, students realized that theoretical knowledge can be better connected with clinical application through the novel teaching mode, so they gradually accepted it.

In addition, students indicated that watching micro-video class through the online platform significantly improved the efficiency of self-learning compared with traditional preview lessons. Micro videos are considered most helpful online self-learning materials by students. However, it is not enough to simply convert the traditional lectures to online video, as the long-time teaching video will frequently distract students from the main point. The knowledge points need to be reasonably designed and divided to compress the lengthy teaching lectures into brief and to-the-point micro videos.

High-quality micro-video classes, highly interactive classroom activities, and leading questions provided before class can greatly increase the attraction to students, improve their self-learning efficiency and relieve their anxiety. Therefore, it is necessary to establish a teaching team to jointly participate in the production of micro videos, the design of classroom activities and extracurricular online answering questions, so as to share teachers' pressure and guarantee the quality of course construction. In this teaching reform, we sought to create high-quality and repeatable online resources and flipped classroom activities, which will continue to be applied to pharmacology course in the future. Since theoretical knowledge doesn't be taught repeatedly, teachers' workload is reduced, and they will prefer to this teaching mode.

Furthermore, although the questionnaire results show that most students are more satisfied with the teaching model of flipped classroom based on micro-video class. It is likely that some students prefer passive learning based on teachers' lectures, which explains the low satisfaction score given by some students (5.80%). Therefore, in addition to improving students' learning initiative via increasing the interest of teaching contents and activities, appropriate self-learning supervision and management is also necessary. In this teaching reform, teachers track students' pre-paration before class through the learning management system of the online platform, so as to effectively guide them to learn. In addition, scoring is also incorporated into pre-paration to encourage students self-learning.

In conclusion, flipped classroom based on micro-video class is a beneficial supplement to the traditional teaching model, and is worth applying and popularizing in pharmacology teaching. However, the potential limitations of this study are also existed. The teaching reform has not been implemented in the whole course of pharmacology, and the teaching effect and course quality is also lack of systematic evaluation, such as assessment of clinical competencies, longitudinal follow-up, experts' evaluation and so on. A more complete curriculum and evaluation system needs to be established for pharmacology course in the future.

## Data Availability Statement

The raw data supporting the conclusions of this article will be made available by the authors, without undue reservation.

## Ethics Statement

The studies involving human participants were reviewed and approved by Ethics Committee of Chengdu Medical College. The patients/participants provided their written informed consent to participate in this study.

## Author Contributions

Y-YW, SL, F-YZ, and XM contributed to conception and design of the study. F-YZ implemented the methods of the study in the two classes. XM supervised the execution of the study. QM performed the statistical analysis. Y-YW, SL, QM, Y-XZ, and SY contributed to the design of teaching content and micro class video. F-LL and F-YZ wrote the first draft of the manuscript. Y-YW and SL wrote the final manuscript. All authors contributed to manuscript revision, read, and approved the submitted version.

## Funding

This work was supported by the Teaching Reform Project of Chengdu Medical College (JG201933, JG201615), Blended learning Teaching Mode Reform Project of Chengdu Medical College and Sichuan Provincial First-class Curriculum Construction Project.

## Conflict of Interest

The authors declare that the research was conducted in the absence of any commercial or financial relationships that could be construed as a potential conflict of interest.

## Publisher's Note

All claims expressed in this article are solely those of the authors and do not necessarily represent those of their affiliated organizations, or those of the publisher, the editors and the reviewers. Any product that may be evaluated in this article, or claim that may be made by its manufacturer, is not guaranteed or endorsed by the publisher.
